# Targeting Farnesoid X Receptor in Tumor and the Tumor Microenvironment: Implication for Therapy

**DOI:** 10.3390/ijms25010006

**Published:** 2023-12-19

**Authors:** Miljana Nenkov, Yihui Shi, Yunxia Ma, Nikolaus Gaßler, Yuan Chen

**Affiliations:** 1 Section Pathology of the Institute of Forensic Medicine, Jena University Hospital, Friedrich Schiller University Jena, Am Klinikum 1, 07747 Jena, Germany; miljana.nenkov@med.uni-jena.de (M.N.); yunxia.ma@med.uni-jena.de (Y.M.); nikolaus.gassler@med.uni-jena.de (N.G.); 2 California Pacific Medical Center Research Institute, Sutter Bay Hospitals, San Francisco, CA 94107, USA; yihui.shi@sutterhealth.org

**Keywords:** FXR, tumor, TME, immunotherapy, combination therapy

## Abstract

The farnesoid-X receptor (FXR), a member of the nuclear hormone receptor superfamily, can be activated by bile acids (BAs). BAs binding to FXR activates BA signaling which is important for maintaining BA homeostasis. FXR is differentially expressed in human organs and exists in immune cells. The dysregulation of FXR is associated with a wide range of diseases including metabolic disorders, inflammatory diseases, immune disorders, and malignant neoplasm. Recent studies have demonstrated that FXR influences tumor cell progression and development through regulating oncogenic and tumor-suppressive pathways, and, moreover, it affects the tumor microenvironment (TME) by modulating TME components. These characteristics provide a new perspective on the FXR-targeted therapeutic strategy in cancer. In this review, we have summarized the recent research data on the functions of FXR in solid tumors and its influence on the TME, and discussed the mechanisms underlying the distinct function of FXR in various types of tumors. Additionally, the impacts on the TME by other BA receptors such as takeda G protein-coupled receptor 5 (TGR5), sphingosine-1-phosphate receptor 2 (S1PR2), and muscarinic receptors (CHRM2 and CHRM3), have been depicted. Finally, the effects of FXR agonists/antagonists in a combination therapy with PD1/PD-L1 immune checkpoint inhibitors and other anti-cancer drugs have been addressed.

## 1. Introduction

Cancer is a major public health problem and the second leading cause of death worldwide, behind only cardiovascular diseases [1,2]. In 2023, 1,958,310 new cancer cases and 609,820 cancer deaths are projected to occur in the United States [3]. During the past two decades, the treatment paradigm for cancer has been largely changed due to two major revolutions: targeting actionable genetic alterations and immuno-oncology. The implementation of personalized therapy, together with better access to diagnostics in primary care, leads to an improved clinical outcome [4,5]. However, the overall survival for many types of cancer is still poor. Thus, the identification of oncogenic pathways and molecular targets for achieving early diagnosis and developing new anti-cancer drugs is essential for cancer management.

Bile acids (BAs), known as detergents involved in the digestion of lipids, are soluble derivatives of cholesterol that, subsequently, undergo bacterial transformation, yielding a diverse array of metabolites [6]. BAs binding to their receptors activate signaling pathways involved in the regulation of metabolic disorders, the immune response, and carcinogenesis [7,8]. BA receptors mainly include membrane receptors like TGR5 (also called G protein-coupled bile acid receptor 1 GPBAR1) and sphingosine-1-phosphate receptor 2 (S1PR2), as well as muscarinic receptors (CHRM2 and CHRM3) and nuclear receptors (NRs) such as the farnesoid X receptor (FXR, also known as NR1H4), vitamin D receptor (VDR, NR1H1), pregnane X receptor (PXR, NR1H2), liver X receptor (LXR), constitutive androstane receptor (CAR, NR1H3), and small heterodimer partner (SHP). FXR is one of the well-characterized BA receptors. FXR was first named in 1995 due to its activation by supraphysiological levels of farnesol [9]. Later, it was recognized to regulate the primary BA synthesis initiated by cholesterol 7α-hydroxylase (CYP7A1) in the liver [10,11]. FXR is expressed in many organs and dysregulated in various types of cancer [12]. FXR also exists in immune cells, which are the major components of the tumor microenvironment (TME) [13,14]. In response to the alteration of BAs, a series of FXR activities could be triggered by immune cells. Therefore, determining the function of FXR in both cancer cells and the TME may have immense value for tumor therapy. Indeed, many studies have provided evidence that FXR participates in cancer progression and development through distinct mechanisms, and its agonists and antagonists enhance or inhibit tumor cell growth solely or in combination with chemotherapy, targeted therapy, or immunotherapy. This implies that the modulation of FXR may have vast therapeutic potential in cancer treatment. However, challenges still remain regarding the development of FXR-based therapeutic strategies.

## 2. Bile Acid Synthesis

Bile acids (BAs) are amphipathic molecules with a steroid structure, known for their role in regulating fat digestion and the absorption of liposoluble vitamins, owing to their strong detergent properties [15,16]. Along with their well-known function as emulsifiers, BAs are very important signaling molecules in regulating various processes including glucose, lipid metabolism, cholesterol homeostasis, and immunity [16,17]. BAs are synthesized mostly in the liver, secreted and concentrated in the gallbladder, and, upon meal uptake, released into the small intestine [18]. Two BA synthesis pathways have been described: classical (neutral) and alternative (acidic) [19]. The classical pathway starts by converting the cholesterol backbone to a more hydrophilic structure of 7α hydroxy cholesterol, mediated by enzyme cholesterol 7α hydroxylase (CYP7A1). CYP7A1 is a member of the cytochrome P450 family and is a crucial enzyme for cholesterol metabolism and a rate-limiting enzyme for BA synthesis in the liver [20]. CYP7A1 is expressed in the endoplasmic reticulum (microsome) of the hepatocytes [21]. Next, 7α-hydroxycholesterol is converted to 7α-hydroxy-4-cholesten-3-one (C4), by hydroxysteroid dehydrogenase 3B7 (HSD3B7) [21]. C4 is also described as a surrogate serum marker for BA synthesis [21,22,23]. In the presence of sterol 12α-hydroxylase (CYP8B1), C4 is directed towards cholic acid (CA) synthesis, whereas, in the absence of CYP8B1, C4 is directed towards chenodeoxycholic (CDCA) acid synthesis [21]. Trihydroxycholestanic acid (THCA) and dihydroxycholestanic acid (DHCA) are products of a reaction mediated by mitochondrial sterol 27-hydroxylase (CYP27A1). THCA and DHCA are activated by bile acid-CoA synthetase (BACS, long-chain acyl-CoA synthetase, SLC27A5) and transferred into peroxisomes, where the formation of cholyl-CoA and chenodeoxycholyl-CoA occurs [21]. These bile acyl-CoAs are conjugated with the amino acids glycine (G) or taurine (T) by the enzyme bile acid-CoA amino acid N-acyltransferase (BAAT) [21,24]. Glyco/tauro cholic acid and glyco/tauro chenodeoxycholic acids formed through conjugation show increased solubility at their physiological pH to form sodium salts, and BAs are stored in the gallbladder until meal uptake stimulates the release of bile [21]. The alternative BA synthesis pathway is initiated by sterol 27-hydroxylase (CYP27A1) to form 25(R)-26-hydroxycholesterol, and the intermediates of the alternative pathway are shuttled in the cycle of chenodeoxycholic (CDCA)-bile acid production [19,21,25].

Glyco/tauro cholic acid and glyco/tauro chenodeoxycholic acids are excreted through the bile salt export pump (BSEP) into the gall bladder [21]. BAs are released into the upper intestine after food uptake. Most BAs (95%) are actively reabsorbed by enterocytes in the terminal ileum via the apical sodium-dependent bile acid transporter (ASBT) [21]. In enterocytes, BAs bind to the ileal bile-acid-binding protein (IBABP), which shuttles them to the organic solute transporter α and β (OSTα and OSTβ) heterodimers [24]. Subsequently, BAs are excreted into the portal blood circulation [23]. Returning to the liver, BAs are taken up by the hepatic sodium taurocholate co-transporting polypeptide (NTCP) [21]. The enterohepatic circulation of BAs is highly efficient in maintaining a consistent total BA pool size and composition in the gastrointestinal system [21]. 

**Figure 1:**
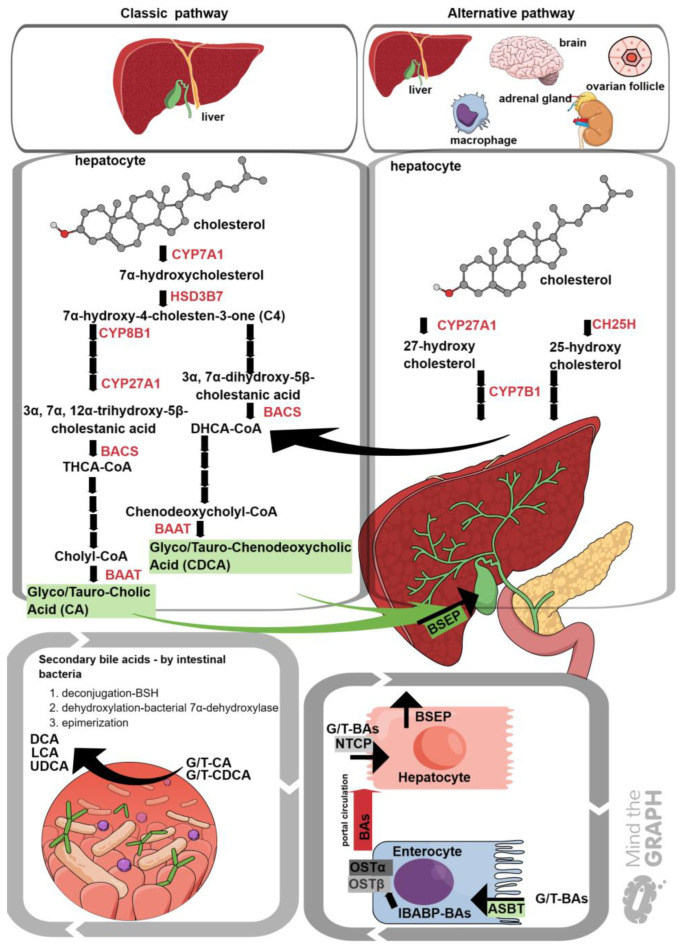
Synthesis of bile acids via the classic (neutral) and alternative (acidic) pathways. The classic pathway takes place in the liver and is initiated by cholesterol conversion to 7α hydroxycholesterol mediated by cholesterol 7α hydroxylase (CYP7A1) [21,25]. The alternative pathway occurs in both the liver and extrahepatic tissues including the brain, adrenal glands, macrophages, and ovarian follicles [21,29]. The alternative pathway is initiated by the conversion of cholesterol to 25(R)-26-hydroxy cholesterol (27-hydroxycholesterol) metabolized by sterol 27-hydroxylase (CYP27A1), 25-hydroxy cholesterol produced by sterol 25-hydroxylase (CH25H), and 24-hydroxycholesterol catalyzed by sterol 24-hydroxylase (CYP46A1) in the brain [30]. Secondary bile acids such as DCA, LCA, and UDCA are formed from intestinal bacterial deconjugation/dehydroxylation/7β epimerization [31]. The secondary bile acids are absorbed and returned to the liver through enterohepatic circulation. Detailed information on BA biosynthesis can be found in the text. The figure was generated using Mind the Graph. CYP7A1: cholesterol 7α hydroxylase, HSD3B7: hydroxysteroid dehydrogenase 3B7, C4: 7α-hydroxy-4-cholesten-3-one, CYP8B1: Sterol 12α-hydroxylase, CYP27A1: sterol 27-hydroxylase, BACS: bile acid-CoA synthetase, BAAT: bile acid-CoA amino acid N-acyltransferase, CDCA: chenodeoxycholic acid, CA: cholic acid, CH25H: 25-hydroxylase, CYP7B1: 25-hydroxycholesterol 7-alpha-hydroxylase, DCA: deoxycholic acid, LCA: lithocholic acid, UDCA: ursodeoxycholic acid; BSEP: bile salt export pump, NTCP: sodium taurocholate co-transporting polypeptide, ASBT: apical sodium-dependent bile acid transporter, OSTα/β: organic solute transporter alpha/beta, IBABP: ileal bile-acid-binding protein.

Two major primary BAs are chenodeoxycholic acid (CDCA) and cholic acid (CA). Conjugated BAs not actively reabsorbed in the ileum are subjected to deconjugation, dehydroxylation, and epimerization by bacteria, mainly in the colon [14,26]. Deconjugation is mediated by bacterial bile salt hydrolase (BSH), followed by the 7-dehydroxylation of CA and CDCA catalyzed by bacterial 7α-dehydroxylase to form secondary BAs deoxycholic acid (DCA) and lithocholic acid (LCA), respectively [26]. Ursodeoxycholic acid (UDCA) is a secondary BA, produced through the 7β epimerization of CDCA [27,28]. The intestinal microbiota contributes to other BAs such as the 3-, 7-, and 12-oxo-bile acids in the colon [14]. The synthesis of bile acids via the classic (neutral) and alternative (acidic) pathways is illustrated in Figure 1.

## 3. Bile Acid Receptors

Tightly controlling the enzymes and transporters involved in BA synthesis is critical for BA homeostasis and is mediated by BA signaling [32]. BAs bind to receptors and activate the downstream signaling pathways [33,34]. FXR is a nuclear receptor that is highly expressed in enterocytes and hepatocytes. It is involved in the regulation of BA synthesis, uptake, secretion, and detoxification, regulating intestinal FGF15/19 signaling and immune response via the inhibition of NF-kB in the liver and intestine [33,34,35,36]. CDCA has the highest affinity for FXR receptor binding, followed by DCA, LCA, and CA [34]. TGR5 is a membrane-bound receptor expressed in the epithelial cells, immune cells, and intestinal nerves in the gut and biliary tract [33,34]. It plays a role in inhibiting the pro-inflammatory function of macrophages by downregulating TLR4-mediated NF-kB signaling, as well as glucose-metabolism-related functions [33,34,37,38]. LCA is the most potent agonist of TGR5, followed by DCA, CDCA, and CA [34]. The vitamin D receptor is a nuclear hormone receptor expressed in intestinal epithelial cells and exerts immunomodulatory and anti-inflammatory functions [34,39,40]. LCA is a vitamin D receptor agonist [33]. Retinoid-related orphan receptor γt (RORγt) is a nuclear hormone receptor expressed in T helper 17 (Th17) cells, ILC3, and γδT cells, and is involved in regulating Th17 cell differentiation [33,34,41]. Its agonists are 3oxo-LCA and isoLCA [34]. Sphingosine-1-phosphate receptor 2 (S1PR2), a membrane-bound receptor, is expressed mostly in intestinal epithelial cells and hepatocytes with a binding affinity towards GCA, TCA, GCDCA, and TCDCA [34]. S1PR2 plays a role in insulin signaling through ERK 1/2 /MAPK/AKT activation [33,42]. The constitutive androstane receptor (CAR) is a nuclear hormone receptor expressed in hepatocytes and is important in phase I and II drug detoxification [33,34,43,44]. LCA is an endogenous bile acid agonist of CAR [33]. The pregnane X receptor (PXR), a nuclear hormone receptor expressed in intestinal epithelial cells and hepatocytes, regulates drug detoxification and elimination. LCA and its 3-keto oxidized form are agonists of PXR [34,43].

## 4. Farnesoid X Receptor (FXR) Structure

*FXR* has two genes, *FXRα* (*NR1H4-nuclear receptor subfamily 1 group H member 4*) and *FXRβ* (*NR1H5-nuclear receptor subfamily 1 group H member 5*, pseudogene). The FXR gene is located on chromosome 12q23.1, and encodes four different transcripts including *FXRα1*, *FXRα2*, *FXRα3*, and *FXRα4*. *FXRβ* is a pseudogene in humans [45]. *FXR* is not only expressed in the intestine and liver, but also in other tissues such as the heart, kidney, thymus, spleen, and vasculature [46]. FXR belongs to the steroid-analog nuclear receptors that regulate downstream transcriptional processes mainly by forming heterodimers with the retinoid X receptor family (RXR) [47]. Compared to other steroid receptors, BAs bind the ligand-binding pocket of FXR, facing their A rings to the AF2 (activation function domain 2) helix at their physiologic concentrations over a wide range (10–100 µM) [48]. This wide range of concentrations indicates that the FXR receptor has a lower affinity and is associated with decreased specificity [48]. This suggests that FXR might be activated by a wide range of additional compounds [48]. From all natural ligands, CDCA has the highest potential to activate FXR [49]. Like other nuclear receptors, FXR has four functional domains: a ligand-independent transcriptional activation domain (AF1), a core DNA-binding domain (DBD), a C-terminal ligand-binding domain (LBD), and a ligand-dependent activation function domain (AF2) [50]. AF2 is crucial because its stabilization and binding to the core of the LBD creates a surface for co-activator binding [51]. FXR has been reported to have four isoforms, FXRα1 (+), FXRα1 (−), FXRα2 (+), and FXRα2 (−), generated by alternative promoter usage and splicing [52]. Both FXRα1 and FXRα2 are the result of alternative promoter usage, exon1 or exon3, respectively. The amino acid sequence and size of FXRα1 (+) (476 aa) differ in the sequence (1–36 aa) compared to FXRα2 (+) (486 aa) [53]. (FXRα1 (−) (472 aa) and FXRα2 (−) (482 aa) are lacking in the 12 bp insert (MYTG; 207–210 aa) produced as a result of alternative splicing at the end of exon 5 [53]. Vaquero et al. reported differences in the expression patterns of *FXRα1* (*±*) and *FXRα2* (*±*) in different tissues, with a predominance of *FXRα1* in the liver and *FXRα2* in the intestine [53,54]. Unconjugated BAs have a higher potential to activate FXR than conjugated BAs do [53,54]. CDCA induces the highest upregulation of downstream FXR target genes including *BSEP*, *SHP*, *OSTβ*, and *TCEA2* [55]. The fold of upregulation of *BSEP* is dependent on the activated FXR isoform, following the order FXRα1 (−) > FXRα2 (−) > FXRα1 (+) > FXRα2 (+); for SHP, it was FXRα1 (−) = FXRα2 (−) > FXRα1 (+) = FXRα2 (+), whereas the four isoforms display similar efficiencies in stimulating *OSTβ* [55]. The ratio of FXRα1 (±) to FXRα2 (±) differs among different cell types, but all four isoforms are always present with different ratios [55]. Among the four isoforms, FXRα1 (−) induces the highest upregulation of FXR target genes upon agonist stimulation. The presence of cell- and tissue-specific cofactors is the main determinant of FXR binding to specific groups of genes.

Lysine residues within human FXR are highly conserved, and their replacement could influence subcellular localization, protein–protein association, and protein-DNA binding. As described by Sun et al., replacing lysine with arginine at the following positions (K122, K210, K229, and K460) of FXR alters the expression of FXR targets including OSTα/β) and BSEP in a ligand-dependent manner. Furthermore, the mutation of K210R may affect FXR heterodimerization with RXR and reduce protein-to-DNA interaction at the hBSEP promoter [56].

## 5. Post-Translational Modifications of FXR

### 5.1. Sumoylation

The sumoylation of proteins is a reversible post-translational modification which is characterized by the covalent attachment of small ubiquitin-like modifiers (SUMOs) mediated by three-step enzyme cascade reactions including activation, conjugation, and ligation [57]. The SUMO modification of proteins is mediated by the SUMO-activating enzyme, E2 enzyme Ubc9, and E3 enzyme-ligases PIAS1-4 (the protein inhibitor of activated signal transducer and activator of transcription 1 to 4), resulting in isopeptide bond formation between the glycine of the C-terminal domain of SUMO and a lysine from the target protein [58]. SUMO1 is structurally similar to ubiquitin and covalently attached to certain lysine residues of specific target proteins [59]. Sumoylation influences several aspects of target proteins, including subcellular localization, dimerization, DNA binding, and activity [59]. The sumoylation of transcription factors results in both transcriptional activation and repression [60]. Sumo consensus sites have been detected in AF1 and the ligand-binding domains of FXR. Balasubramaniyan et al. reported sumoylation at lysine 122 and 275 located on AF1 of FXR in the liver by SUMO1 [58]. The effect of FXR sumoylation was investigated both in vitro (hepatocytes) and in vivo (mouse liver) [58]. The expression of Sumo1 markedly inhibited the ligand-dependent transactivation of BSEP and SHP promoters by FXR/retinoid X receptor α (RXRα) in HepG2 cells [58]. Mutation at the known SUMO consensus sites at lysines 122 and 275 of FXR completely prevented sumoylation [58]. Sumoylation did not affect the nuclear localization of FXR [58]. Sumo1 expression did not influence the transactivation of the *BSEP* and *SHP* promoters by an FXR construct with combined Lys122, Lys275, and Glu277 mutations [58]. Furthermore, the enhanced recruitment of SUMO proteins to the promoters of *BSEP* and *SHP* may attenuate the expression of these genes in cholestasis, indicating an important role of SUMO in cholestatic disease [61]. Vavassori et al. demonstrated that sumoylation at lysine 277, located on the ligand-binding domain of FXR, was essential for retaining FXR full trans-repression of *TNF-α* gene expression, suggesting that the sumoylation of FXR may be required for FXR-mediated trans-repression. FXR has a regulatory role in macrophages when activated by its ligands, and it represses the expression of some *toll like receptor 4* (*TLR-4*) regulated genes, as well as proinflammatory cytokines, chemokines, and their receptors [61].

### 5.2. Acetylation

The acetylation of FXR plays an intricate role in regulating gene expression. As demonstrated by Kamper et al., the acetylation of FXR occurs at lysine 217 (K217) within the hinge domain and at lysine 157 (K157) by p300 (acetylase) [62]. Interestingly, they showed that mutations within these two sites led to decreased FXR stability but increased heterodimerization with RXRa, DNA binding, and transactivation activity [62]. Furthermore, the silencing of SIRT1 (deacetylase) in mouse liver was associated with increased levels of endogenous acetylated FXR, indicating that SIRT1 alters FXR acetylation in vivo. In normal mice without any metabolic abnormalities, FXR is activated by ligand binding and recruits p300 to promoter sites of the downstream genes, accompanied by SIRT1 dissociation. The acetylation of histones by p300 is correlated with the increased activation of FXR target genes. However, the acetylation of FXR limits its transcriptional activity. This seemingly contradictory event could be explained by normal physiological conditions where the acetylation/deacetylation of FXR is tightly regulated by p300 and SIRT1 to terminate the response to stimulus [63]. In mice with metabolic disease, a constant increase in the acetylation level of FXR prevents the heterodimerization of FXR/RXRα, as well as the DNA binding of FXR/RXRα, leading to a reduction in the transactivation of FXR metabolic target genes. It has been speculated that the dynamic acetylation and deacetylation of FXR in normal mice are important for the activation of metabolic target genes, whereas in mice with metabolic diseases, constantly elevated acetylation blocks the transactivation of these genes [62]. Acetylation at position (K217) of FXR was found in obese mice [64]. This was accompanied by the dysregulated expression of pro-inflammatory cytokines, macrophage infiltration, and increased triglyceride levels, resulting in further deterioration of the metabolic inflammation [64]. This post-translational modification prevented the SUMO2 modification of FXR at K277 by inhibiting the SUMO ligase PIASγ [64]. FXR modified by SUMO2 has been found to interact with NF-κB, precisely through trans-repression, leading to the downregulation of inflammatory gene expression. FXR/RXRα interaction is blocked through this modification; however, FXR/RXRα downstream target genes are not affected [64].

### 5.3. Methylation

The acetylation and methylation of the histone tail have been recognized to elevate the accessibility of genes to transcription factors and the basal transcriptional activity. The direct methylation of FXR regulates the expression of FXR target genes [65,66]. In addition to the methylation of histones at the loci of FXR target genes, FXR itself might also be modified by coactivators, as previously reported for acetylation. Both an in vitro and in vivo study show that the methylation of FXR occurs within its hinge domain at lysine (K206) and is mediated by the methyltransferase SET7/9, known as histone H3K4 monomethyl-transferase [67]. The methyation of FXR by SET7/9 also significantly enhanced the transactivation of the *SHP* and *BSEP* promoters, whereas the methylation of FXR with the mutation at lysine (K206) failed to do so. The disruption of histone by lysine methyl transferase MLL3 is observed in obstructive cholestasis [68].

### 5.4. Phosphorylation

The activity of FXR can also be modulated by phosphorylation. It is found that phosphorylation at serine S135 and S154 in the DNA-binding domain of FXR is essential for full ligand-dependent transcriptional activation. The phosphorylation of FXR by PKCα enhances its transcriptional activity by increasing the recruitment of cofactors such as peroxisome proliferator-activated receptor γ coactivator α [69]. These findings indicate that the PKCα-induced phosphorylation of FXR directly modulates the ability of the agonists to activate FXR [69].

## 6. FXR and Immunity

**Figure 2:**
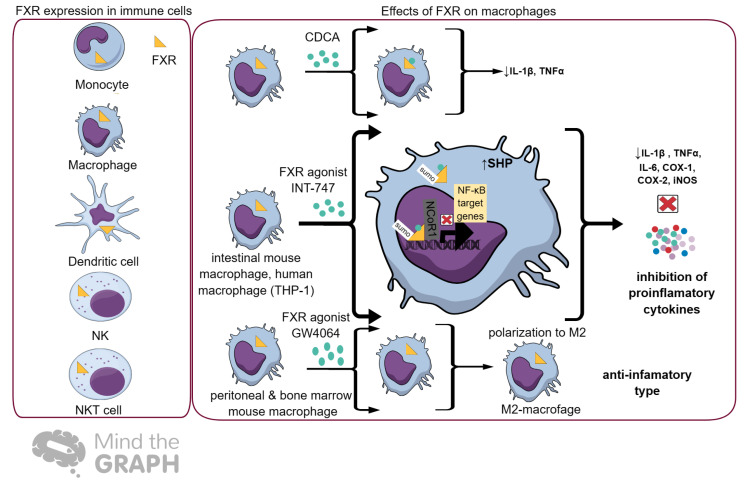
Effects of FXR on macrophages. *FXR* is highly expressed in innate immune cells including monocytes, macrophages, dendritic cells, NK cells, and NKT cells [14]. FXR activation induces itssumoylation in macrophages resulting in the inhibition of pro-inflammatory cytokine secretion by affecting SHP (co-repressor for several cytokines such as IL1β and TNFα), stabilizing NCoR1, and preventing NF-KB binding [61,73,74]. Additionally, FXR activation by GW4064 induces a switch in macrophage polarization towards the M2-anti-inflamatory type [75]. The figure was generated using Mind the Graph. CDCA: chenodeoxycholic acid, NCoR1: nuclear receptor co-repressor 1, SHP: small heterodimer partner, SUMO: Sumoylation.

Nuclear receptors (NRs) of BAs, including *FXR*, *CAR*, and *PXR*, are expressed in human immune cells such as CD4 T cells, CD8 T cells, CD19 B cells, CD14 monocytes, macrophages, dendritic cells. and peripheral blood mononuclear cells [70]. The membrane receptor *TGR5* is also expressed in monocytes and macrophages [14]. The release of pro-inflammatory cytokines, such as *TNFα* and *IL-1* during acute immune responses, negatively regulates the expression of FXR, CAR, and PXR [70,71]. Additionally, NRs inhibit CYP7A1 (cholesterol 7α-hydroxylase), leading to cholesterol accumulation [70,72]. Cholesterol through LXR and BAs through FXR affect T-cell-mediated immune responses. The regulatory role of FXR in innate immunity and homeostasis was described by Vavassori and his colleagues. Colonic inflammation in mice was enhanced in the absence of FXR, leading to the progression of immune-mediated disease. When a synthetic FXR ligand 6-ethyl chenodeoxycholic acid (6E-CDCA) was supplied to mice with colonic inflammation, the inhibitory regulation of NF-κB-dependent genes (*TNF-α*, *IL-1β*, *IL-6*, *COX-1*, *COX-2*, and *iNOS*) and induction of *SHP* were observed, resulting in disease weakening. The activation of FXR by synthetic agonists directs FXR to the promoters of *IL-1β* and *iNOS*, contributing to the stabilization of the nuclear co-repressor on the promoters [61]. FXR affects macrophage activation in the liver and intestine [73,74]. Proinflammatory M1 macrophages are important for initiating adaptive immunity and tissue inflammation [75]. M1 macrophages can cause tissue damage and alter the wound-healing process. In contrast, M2 macrophages regulate tissue regeneration and remodeling [75]. In a study by Jaroonwitchawan et al., an FXR agonist, GW4064, applied both in vitro and in vivo, promoted the polarization into M2 macrophages, along with the upregulation of the retinoic acid pathway [75]. The inhibition of retinoic acid resulted in the suppression of the FXR-mediated polarization of M2 macrophages [75]. Thus, GW4064 treatment may be a therapeutic strategy to promote M2-macrophage-mediated tissue remodeling and repair [75]. Significant effects of FXR in the immune cells within so-called “classical BA-target” organs including the intestine and liver have extensively been elaborated. The noteworthy mechanisms underlying FXR-deficiency-mediated inflammation and fibrogenesis in the lung have been depicted by Murray et al. [76]. FXR is expressed in alveolar epithelial cells, resident alveolar macrophages, pulmonary endothelial cells, and lung fibroblasts [76]. In vivo studies conducted on rats exposed to nitrogen mustard (NM) showed decreased FXR expression, the accumulation of oxidized lipids in lung macrophages, and the formation of macrophage foam cells, leading to pulmonary fibrosis [76]. Targeted deletion of the *FXR* gene in mice supports its key role in regulating both pro- and anti-inflammatory macrophage activation following NM-induced lung injury and oxidative stress [76]. The loss of FXR results in increased numbers of pro-inflammatory/cytotoxic M1 macrophages in the lungs in response to NM-induced injury [76]. Campbell and his colleagues demonstrated that FXR functioned in effector T cells to promote alternative physiological responses to decreased nutrients [77]. The deletion of *FXR* in T cells prevented the starvation-induced loss of lymphocytes and increased effector T-cell fitness under nutrient-limiting conditions [77]. The main effects of FXR on macrophages are illustrated in Figure 2.

## 7. Farnesoid X Receptor (FXR) in Cancer

### 7.1. Hepatocellular Carcinoma (HCC)

FXR is considered a master regulator of hepatic triglyceride and glucose homeostasis [78]. In the study by Yang et al., using an FXR-null mice model, they showed that, in addition to impaired BA homeostasis and abnormal glucose and lipid metabolism, both male and female mice spontaneously develop hepatocellular carcinoma (HCC) between 12 and 15 months of age [79,80]. This indicates a protective role of FXR in HCC. In SK-Hep-1 human hepatoma cells, ectopic expression of FXR leads to a decreased cell proliferation, migration/invasion, and tumor growth, while the inhibition of the FXR expression by miRNA-382-5p promotes the progression of HCC cell lines HepG2 and Huh-7 [81,82]. FXR acts as a tumor suppressor in HCC possibly through the following mechanisms: (1) the suppression of ROX generation, thereby decreasing the risk of DNA damage and genomic instability; (2) the upregulation of its downstream tumor suppressors; (3) reducing liver inflammation and fibrosis by the upregulation of fibrosis-promoting proteins such as collagen, TNF-α, IL-1β, IL-6, MMPs, tissue inhibitor of metalloproteinases 2 (TIMP-2), and transforming growth factor (TGF)-β1; (4), maintaining the normal liver metabolism of BAs, glucose, and lipids [83,84,85]. However, the activation of FXR by its agonists CDCA and GW4064 could lead to enhanced (TGF)-β-induced epithelial-mesenchymal transition (EMT) in HCC cells, implying its potential in promoting tumor metastasis [86]. The discrepancy might be explained by the different activation status of FXR and distinct roles it plays in the early and late stage of tumor development. In human HCC tissue samples, decreased expression of FXR is observed in comparison to normal liver tissues, and the downregulation of *FXR* is significantly correlated with tumor stage and differentiation [87,88]. Additionally, *FXR* overexpression was significantly associated with a more favorable clinical outcome in patients with HCC [89].

### 7.2. Biliary Tract Cancer (BTC)

Biliary tract cancer (BTC) frequently arises under certain cholestatic liver conditions. The intrahepatic accumulation of BAs may facilitate co-carcinogenic effects due to the stimulation of bile duct proliferation, enhancement of inflammation, and reduction of FXR-dependent chemoprotection [90]. FXR is generally expressed on the surface of the bile duct. In human BTC tissues, FXR was downregulated compared to surrounding normal liver tissue and it inhibits BTC cell metastasis by suppressing interleukin-6 mediated EMT [91]. In the nude mouse model, the administration of the FXR activators CDCA and GW4064 not only resulted in a significant inhibition of tumor growth but also effectively blocked the growth-stimulatory effect caused by glycodeoxycholic acid (GDCA), a conjugated BA [92]. It is well-known that miRNAs, as epigenetic modulators, modulate their target genes without altering the gene sequences [93]. FXR was downregulated by oncogenic miRNA-421, leading to increased cell proliferation, colony formation, and migration in BTC [94]. The inhibition of FXR by shRNA could significantly block the GCDC-induced metastasis of BTC cells, indicating that targeting FXR may suppress tumor metastasis [95]. Clinically, the low expression of FXR is linked to tumor growth and the poor survival of the BTC patients [96].

### 7.3. Colorectal Carcinoma (CRC)

Besides high-fat diets, obesity, and diabetes, elevated serum levels of toxic BAs are significantly correlated to the development of CRC [35,97]. In human normal intestinal mucosa, a high expression level of FXR is found in well-differentiated surface epithelia [98]. FXR is dramatically decreased from the terminal ileum, a part of the gastrointestinal tract with a rare incidence of carcinoma, to the distal parts of the colon, where carcinoma frequently occurs [99]. Basically, FXR exerts an anti-tumor effect in CRC. FXR-null mice exhibit enhanced intestinal epithelial cell proliferation and tumor progression [100]. FXR inhibits intestinal carcinogenesis in both APC^Min/+^ mice and a bowel inflammatory mouse model through the inactivation of the Wnt/β-catenin signaling and induction of apoptosis [98]. Similarly, Smith et al. reported that the activation of FXR by sodium taurocholate inhibited intestinal adenoma formation in APC^Min^/^+^ mice [101]. A crosstalk between FXR and EGFR was proposed based on the observation that blocking FXR activity with guggulsterone, an FXR antagonist, stimulated time- and dose-dependent EGFR (Tyr845) phosphorylation and ERK activation, while the activation of FXR by its agonist GW4064 attenuated cell proliferation by the inactivation of the EGFR/ERK signaling pathway [102]. Recently, evidence has shown that FXR regulates intestinal cancer stem cell proliferation. The selective activation of intestinal FXR restricts the abnormal growth of Lgr5+ cells which mediate the key adenoma-to-adenocarcinoma transformation, thereby curtailing CRC progression [103]. More recently, it is found that the activation of FXR and the inhibition of EZH2, a histone-lysine N-methyltransferase, synergistically inhibit CRC through accelerating the FXR nuclear location and the upregulation of caudal-related homeo-box 2 (CDX2) expression [104]. Clinically, the FXR protein is found to be downregulated in human CRC patient samples and the diminished FXR expression is associated with an advanced CRC stage and an adverse prognosis [105]. On the mRNA level, *FXR* is reduced in colon adenomas and even more profoundly reduced in colon adenocarcinomas compared to normal colonic tissues [106]. The epigenetic mechanism is partially responsible for the gene silencing of *FXR*. The *FXR* promoter is methylated in ~12% CRC, and the inhibition of DNA methylation and KRAS silencing both increased FXR expression [105,106]. In CRC samples, an inverse correlation was detected between the expression of FXR and miR-192-3p, an upstream suppressor of FXR [52]. Moreover, FXR can be regulated by transcription factors. For example, CDX2 binds to the promoter of FXR and facilitates its expression [107]. Commensurate reductions in FXR and CDX2 resulting from inactivating APC mutations are observed in the tumor tissues of Apc^min/+^ mice and familial adenomatous polyposis (FAP) patients, indicating that mutations in CRC-related genes also contribute to intestinal FXR gene silencing [107].

### 7.4. Esophageal and Gastric Cancer

Considerable evidence indicates an association of BA exposure and BA/FXR signaling with esophageal cancer [108]. The inhibition of FXR by FXR shRNA or guggulsterone, an antagonist of FXR, suppressed tumor cell viability and induced apoptosis in vitro and reduced tumor growth in nude mouse xenografts, indicating an oncogenic function of FXR [109]. On the contrary, Feng et al. pointed out that the activation of FXR by GW4064 suppresses esophageal squamous cell carcinoma through antagonizing ERK1/2 signaling pathway [110]. Clinically, FXR is significantly overexpressed in Barrett’s esophagus, and the overexpression of FXR in esophageal adenocarcinoma was associated with a higher tumor grade, greater tumor size, and lymph node metastasis [109,111].

Gastric cancer (GC) is the final outcome of a cascade of pathological alterations including chronic gastric inflammation, intestinal metaplasia (IM), atrophic gastritis, dysplasia, and neoplasia [112]. Abnormal BA signaling participates in the gastric pathogenesis. Bile reflux leads to inflammation of the stomach lining and the activation of FXR promotes intestinal metaplasia of gastric cells with the upregulation of CDX2 [113]. In line with this, Wang et al. depicts that the activation of the FXR/NF-κB pathway results in the upregulation of intestinal markers such as CDX2, MUC2, and KLF4, along with the transcriptional activation of *SNAI2* in BA-induced gastric IM cells [114]. FXR-deficient mice were found to be more susceptible to indomethacin-induced gastric ulceration than their wild-type littermates, and the transfection of FXR into gastric adenocarcinoma cells protected against tumor necrosis factor α (TNFα)-induced cell damage, implying its gastroprotective activity in vitro and in vivo [115].

### 7.5. Pancreatic Cancer

BAs enter the pancreas probably by two different ways: either by reflux through the pancreatic duct or systemically by transport through the blood stream to facilitate lipid digestion [116]. The abnormal secretion of BA may result in BA reflux into the pancreatic duct and further to the epithelial cells or acinar cells from which pancreatic adenocarcinoma is derived [117]. Indeed, as one of the important components of BA signaling, abnormal FXR expression was found in pancreatic cancer. A high expression of FXR was significantly related to lymph node metastasis and a worse prognosis in patients with pancreatic ductal adenocarcinoma (PDAC) [118,119,120]. On the contrary, Giaginis et al. found that an elevated FXR expression is associated with lower tumor aggressiveness and a more favorable prognosis in patients with pancreatic adenocarcinoma [121]. THE Downregulation of FXR expression by siRNA inhibited cell proliferation and decreased cell migration and invasion in pancreatic cells via the activation of the NF-κB pathway [122]. Another in vitro study demonstrates that the induction of the FXR/FAK/c-Jun axis by elevated levels of BA increases the tumorigenic potential of pancreatic cancer cells, along with the upregulation of oncogenic MUC4 expression [123].

### 7.6. Breast and Cervical Cancer

FXR was found to be expressed in human breast cancer tissues and cell lines for the first time by Swales and his colleagues in 2006 [124]. FXR could regulate apoptosis and aromatase expression. Later, it was found that the activation of FXR by CDCA increased cell proliferation and activated estrogen receptor (ER) and estrogen in breast cancer cells, which is in line with the clinical founding that FXR overexpression was significantly correlated with the expression of ER and the proliferation marker Ki-67 in ER-positive breast tumors from postmenopausal women [125]. The research data from Absil et al. suggest that FXR may not only affect proliferation but also breast tumor metastasis in the bone. They found that FXR activation by CDCA increased the expression of numerous bone proteins such as RUNX2, OPN, OC, and BSP in the ER-positive breast cancer cell line MCF-7, while FXR inhibition by guggulsterone decreased bone protein expression [126]. In line with it, in breast cancer patients with bone metastases, a high FXR expression was detected [126]. The activation of FXR not only affects the metastatic potential of breast cancer cells, but it also affects the cell cycle. Treatment with the FXR agonist CDCA or GW4064 leads to apoptosis in breast cancer cell lines with distinct phenotypes, MCF-10A (normal), MCF-7 (receptor positive), and MDA-MB-231 and MDA-MB-468 (triple negative), and decreases the proliferation of tamoxifen-resistant MCF-7TR1 cells [127,128]. In line with that, increased FXR levels in bulk breast tumors correlate with a longer patient survival [121,129].

Compared to breast cancer, research data regarding the function of FXR in cervical cancer are not abundant. Huang et al. demonstrated that the overexpression of FXR inhibited cervical tumor proliferation via upregulating the p14ARF-MDM2-p53 pathway in vitro and in vivo [130,131].

### 7.7. Renal Cell Carcinoma (RCC) and Bladder Carcinoma

The kidney participates in BA circulation and the regulation of BA homeostasis. FXR is highly expressed in the kidney, and BA signaling emerged as being important for renal pathophysiology [132]. Recently, attention has been paid to the role of FXR in renal cell carcinogenesis. Fujino et al. found that the growth of renal adenocarcinoma cells was inhibited by FXR siRNA knockdown, along with the activation of p53/p21 signaling, while the growth of normal renal cells was not affected [133]. This might be due to the shifting function of FXR from cell differentiation in normal cells to cell proliferation in renal carcinoma cells [12,134]. In line with the oncogenic role of FXR, Huang et al. observed that FXR promoted cancer cell proliferation, migration, and invasion via modulating CCNE2, a cell cycle regulator, in clear cell renal cell carcinoma (ccRCC) [135]. A multi-omics analysis including transcriptome sequencing, miRNA sequencing, and proteomics using ccRCC tissues reveals that FXR and macrophage activation pathways could be critically involved in the inhibition of the progression of low-risk ccRCC [136]. Huang et al. identified that the expression of FXR was associated with a high diagnostic accuracy in the early stage (stage I) of ccRCC, and moreover, genetic mutations, as well as the DNA methylation of FXR, were significantly linked to a prognosis in ccRCC patients [135].

In bladder carcinoma, FXR inhibits cancer cell migration, adhesion, and angiogenesis through proteasome degradation, VEGF reduction, AMPK activation, and cholesterol biosynthesis inhibition [137,138]. Consistent with the in vitro data, FXR expression was downregulated in human bladder cancer tissues, compared to the adjacent normal tissues, and a higher expression of FXR was significantly associated with a better clinical outcome [138].

### 7.8. Prostate Cancer

Androgens promote the growth of both normal and cancerous cells by binding to and activating the androgen receptor [139]. The activation of FXR by CDCA or GW4064 negatively interferes with enzymes UGT2B15 and UGT2B17, two major determinants of the androgen response in prostate cancer cells, without affecting cell viability [140]. Later, it was found that FXR could inhibit the proliferation of the prostate cancer cell line LNcaP through the upregulation of the tumor suppressor PTEN and the suppression of lipid metabolism by targeting sterol response element binding protein 1 (SREBP1) [141,142]. In primary prostate tumor samples, FXR expression was downregulated on both mRNA and protein levels, compared to adjacent normal tissues [141].

### 7.9. In Other Tumors

FXR is involved in the process of bone differentiation. The activation of FXR by CDCA stimulates the RUNX2-mediated osteoblastic differentiation of bone marrow stroma cells (BMSCs), whereas the inhibition of FXR leads to an adipocyte-like phenotype [143]. In osteosarcoma, the activation of FXR by GW4064 inhibits cell proliferation via the upregulation of the miR-23b-3p/CCNG1 pathway [144]. In non-small-cell lung cancer (NSCLC), FXR has been recognized as a proto-oncogene. FXR knockdown in NSCLC cells inhibited cell proliferation, blocked xenograft tumor growth in nude mice, and delayed the G1/S transition of the cell cycle by the downregulation of CCND1 expression [145].

## 8. FXR in Tumor Microenvironment and Implication for Anti-PD1/PD-L1 Immunotherapy

FXR is expressed in immune cells and regarded as a regulator of inflammation and the immune response in immune-mediated disease [13,14,146]. The pathophysiological role of FXR in modulating liver and gastrointestinal inflammation and innate immunity has been well addressed [14,147,148]. Emerging evidence shows that FXR affects the immune cells in the tumor microenvironment (TME). The composition of the TME varies between tumor types, but hallmark features include immune cells, stromal cells, blood vessels, and the extracellular matrix [148,149]. The response to the immune checkpoint blockade (ICB) can be affected by the composition of the TME. Several markers are related to the response to the ICB; among them, the PD-1/PD-L1 axis is at the forefront of interactions between tumor cells and the TME [150]. PD-1 binding to PD-L1 inhibits the activation of T lymphocytes and enhances the immune tolerance of tumor cells, thereby causing tumor immune escape [151]. The development of immune checkpoint inhibitors, particularly the PD1/PD-L1 inhibitors, has been considered a major progress in oncology in the past decade [152]. Until now, four anti-PD-1 monoclonal antibodies (Abs) including pembrolizumab, nivolumab, cemiplimab, and dostarlimab, as well as three anti-PD-L1 monoclonal Abs containing atezolizumab, durvalumab, and Avelumab, have been approved by the FDA for immunotherapy in solid tumors including melanoma, NSCLC, CRC, HCC, RCC, breast cancer, etc. [153]. PD-L1 expression on tumor cells is one of the important predictive markers for anti-PD1/PD-L1 therapies in various malignancies [154,155]. However, only a subset of cancer patients benefit from anti-PD1/PD-L1 therapy with an objective response rate less than 45% in melanoma, less than 20% in NSCLC, and 20–25% in RCC and CRC [155,156,157,158,159]. Additionally, some of patients initially showing a clinical response acquire resistance after a few years, leading to tumor relapse and progression. Thus, the identification of robust biomarkers for anit-PD1/PD-L1 immunotherapy and the application of combination strategies with backbone immunotherapeutic drugs might improve the clinical outcome. Accumulating evidence demonstrates that FXR builds an immunosuppressive microenvironment in different tumor entities. Moreover, FXR agonists sensitize PD1/PD-L1 immunotherapy in cancer. The influence of FXR on tumor immunity has mainly been studied in lung, liver, colon, breast, and kidney tumors.

### 8.1. Lung TME

You et al. found that FXR could cause immunosuppression by decreasing the proliferation and function of CD8+ T-cells in the FXR^high^PD-L1^low^ NSCLC cell line [160]. This phenomenon was also seen in the Lewis lung carcinoma (LLC) mouse model, where FXR attenuated infiltrating immune cells and constructed an immunosuppressive microenvironment [160]. Additionally, compared to mock LLC tumors, an increased susceptibility to anti-PD-1 treatment in FXR^high^PD-L1^low^ mouse LLC tumors was observed, indicating a predictive value of FXR for lung cancer immunotherapy [160]. In line with that, an inverse relation between FXR and PD-L1 expression was observed in NSCLC patient samples, and the patients with subtype FXR^high^PD-L1^low^ tumors had worse clinical outcome [160]. Tian et al. showed that the upregulation of PD-L1 could be mediated partly by FXR inhibition in NSCLC cells treated with an FXR antagonist Z-guggulsterone, an active compound extracted from the gum resin of the Commiphora mukul tree [161]. In the mouse LLC model, treatment with Z-guggulsterone enhanced PD-L1 expression in a dose-dependent manner [161]. This indicates that the inhibition of FXR may sensitize the immunotherapy with anit-PD1/PD-L1 antibodies, since PD-L1 expression is a predictive biomarker for immunotherapy in NSCLC patients.

### 8.2. Liver TME

In the liver, FXR regulates the activation of NKT cells. *FXR* gene ablation leads to a time-dependent increase of the liver expression of osteopontin, a NKT cell-derived extracellular matrix protein, and immunoregulatory cytokine in a rodent model of acute hepatitis [162]. In HCC, accumulating evidence reveals that BAs affect the tumor immune response and tumor progression mainly through FXR signaling. The inhibition of FXR by norcholic acid upregulates PD-L1 on the surfaces of HCC cells and tumor-derived exosomes, which dramatically dampens the function of CD4+ T cells, resulting in an immunosuppressive tumor microenvironment [163]. In primary HCC patients, a negative correlation between PD-L1 and FXR expression was found, and HCC patients with FXR^low^PD-L1^high^ tumors had an unfavorable prognosis [163]. Moreover, the combination of the FXR agonist GW4064 and an anti-PD-1 antibody achieved a tumor regression in a HCC syngeneic mouse model, implying the potential therapeutic value of an FXR agonist in HCC immunotherapy.

Ji et al. found that the delivery of obeticholic acid (OCA), a modified bile acid derivative that acts on FXR as an agonist, via nanoapproach significantly suppressed hepatic tumor growth in a murine orthotopic H22 tumor mode by increasing anti-tumor immunity [164]. Immunological analysis revealed that the OCA treatment led to the augmented secretion of CXCL16 and IFN-γ, as well as increased NKT cell populations inside the liver tumor, suggesting its potential efficacy in immunotherapy [164].

### 8.3. Colon TME

FXR affects the function of immune cells in inflammatory diseases in the bowel such as Crohn’s disease and ulcerative colitis, which, over time, could develop into CRC [165,166]. In a mouse model of colitis-associated CRC, FXR regulates the recruitment, polarization, and maturation of gut macrophages, and crosstalks with Th17 cells in the TME to suppress tumor progression [167]. The modulatory role of FXR in gut macrophages highlights the potential of FXR as a therapeutic target for CRC. GW4064 induced apoptosis, a blocked cell cycle, the mediated immunogenic cell death of CRC cells, and upregulated PD-L1 expression via the activation of FXR and MAPK pathways in vitro [168]. Furthermore, the combination of PD-L1 Ab with GW4064 increased CD8+ T-cell infiltration and exhibited excellent anti-tumor effects in CT26 xenograft models, with 33% tumor-bearing mice cured [168]. Given the fact that, clinically, less than 25% of CRC patients benefit from anti-PD1/PD-L1 immunotherapy, a combination with the FXR agonist and PD-L1 immune checkpoint blockade might improve the efficacy of immunotherapy in CRC patients. Interestingly, the FXR antagonist UDCA, in combination with anti-PD-1Ab, also shows a stronger anti-tumor effect in a CRC mouse model, accompanied by increased anti-tumor CD8+ T-cell responses, decreased Treg cells among TILs, and enhanced tumor-specific immune memory [169,170]. The reduction of anti-tumor immunosuppression by UDCA is associated with carboxyl terminus of Hsc70-interacting protein (CHIP)-mediated TGF-β degradation [170].

### 8.4. Breast TME

Recently, it has been reported that microbiome-derived BAs are accumulated in the breast TME, resulting in reduced aggressiveness and metastatic potential of the cancer cells and a better clinical outcome in primary breast cancer patients [171]. In line with that, Wu et al. identified that adipocytes and preadipocytes were significantly infiltrated in the microenvironment of breast cancer exhibiting a high BA metabolism, which was associated with a lower level of Ki67, a proliferation marker. Additionally, the BA metabolism was significantly correlated with four micro-organisms including *Anaerococcus*, *Collimonas*, *Gammaretrovirus*, and *Hymenobacter* [172]. FXR is also expressed in human cancer-associated fibroblasts (CAFs) isolated from breast cancer patients. The activation of FXR by GW4064 decreases CAF migration, as well as stress-fiber formation and contractility [129]. The role of FXR in inhibiting the tumor-stimulatory activity of CAFS was also reported by Giordano and his colleagues. They found that FXR exerts this function via the inhibition of the cytokine signaling 3 (SOCS3) pathway [173]. The data indicate the therapeutic potential of the FXR agonist GW4064 targeting both tumor cells and the TME to combat breast cancer.

### 8.5. Kidney TME

The expression of *FXR* is found to be associated with the infiltration of immune cells in ccRCC. Using a comprehensive bioinformatics analysis based on the data published in databases including TCGA and GEO, Huang et al. identified that *FXR* expression was positively significantly correlated to CD4 + T cell, macrophage/monocyte, and neutrophil infiltration levels, while it is negatively associated with CD8 + T-cell infiltration levels [135]. This implies a potential role of FXR in reshaping the TME of ccRCC. Thus, targeting FXR may lead to a decreased level of tumor-associated macrophage infiltration, especially the infiltration of the M2-polarized macrophages, a negative prognostic marker in cancer. The involvement of FXR agonists or antagonists in the immunotherapy of cancer is summarized in Table 1.

## 9. Influence of Other Bile Acid Receptors on the TME and the Implication for Immunotherapy

Besides FXR, several studies revealed that other BA receptors such as TGR5, S1PR2, and CHRM2, as well as CHRM3, affected tumor immunity by encompassing the TME.

### 9.1. TGR5

TGR5 is a membrane BA receptor, mainly activated by LCA and DCA. Ligand binding to the TGR5 receptor leads to the activation of adenylate cyclase, the generation of cAMP, and the activation of downstream MAPK and AKT, as well as PKA, signaling pathways [6,174,175]. TGR5 exerts tumor-suppressive or oncogenic functions in cancer, depending on the cellular context [176]. Recently, the role of TGR5 in the TME has been studied. Using Gene Expression Integration (GTEx), Human Protein Atlas, and The Cancer Genome Atlas (TCGA), Guan et al. performed a pan-cancer analysis to clarify the correlation between *TGR5* expression and immune system infiltration including CD4 T cells, CD8 T cells, T-cell regulators, CAF, and tumor-associated macrophages (TAMs). It turned out that *TGR5* expression in melanoma was negatively significantly linked to the infiltration level of the M2 macrophages, consistent with the observation that *TGR5* overexpression reduced the viability of skin cancer cells [177]. In contrast to its anti-tumor immunity in melanoma, TGR5 is required for the M2 polarization of TAMs, and TGR5 suppresses anti-tumor immunity in NSCLC through the TAM-mediated CD8+ T-cell suppression and the activation of the cAMP-STAT3/STAT6 signaling, implying its onco-immunological role in NSCLC [178]. Correspondingly, the expression of TGR5, together with a high infiltration of TAMs, is associated with a poor prognosis in NSCLC patients [178].

### 9.2. S1PR2

S1PR2 is also a BA cell membrane receptor. It is mainly activated by conjugated BAs to upregulate sphingosine kinase 2 (Sphk2) [179,180]. Through ligand binding, S1PR2 activates the downstream pathways like MAPK/ERK, AKT, and JNK1/2 [181,182]. *S1PR2* is expressed in immune cells including B cells, macrophages, monocytes, and eosinophils/mast cells, and plays a role in immunity [183,184]. Indeed, a pan-cancer analysis based on the data from GTEx and TCGA shows that, in cervical squamous cell carcinoma and endocervical adenocarcinoma patients, the expression of *S1PR2* is markedly positively related to immune cells such as the dendritic cell, neutrophil, CD4+ T cell, and macrophage, as well as B cell, and patients with a lower *S1PR2* expression have a worse prognosis [185]. In pancreatic cancer, the overexpression of SphK1 leads to the secretion of S1P. S1P binds to S1PR2 and induces stromal cells to release MMP-9, which moderates the TME through affecting the immune cell infiltration [186,187]. Additionally, taurocholic acid TCA, a conjugated BA, was found to accelerate the growth of S1PR2-overexpressing pancreatic cancer both in vitro and in vivo, suggesting that S1PR2 may be a potential therapeutic target in cancer [188].

### 9.3. CHRM2 and CHRM3

The cholinergic receptors muscarinic (CHRMs) can be activated by acetylcholine- and taurine-conjugated BAs. They are overexpressed in various types of cancer, promoting or suppressing tumor cell progression and metastasis. For example, CHRM2 protects against pancreatic cancer and colon neoplasia development, while CHRM3 promotes gastric and CRC proliferation and enhances PDAC severity [189]. Moreover, *CHRMs* are expressed in immunocytes and play a role in modulating the TME. A positive correlation between *CHRM2* expression and the immune cell CD8+ T-cell infiltration was found in lung, gastric, and colon, as well as liver cancer, and the *CHRM3* expression was positively significantly related to CD8+ T-cell infiltration in liver and prostate cancer [190].

### 9.4. VDR

Vitamin D receptor (VDR) is expressed in immune cells including dendritic cells, macrophage, B cells, and T cells [191]. Besides vitamin D, VDR can be activated by the secondary bile acid LCA. It has been reported that LCA controls adaptive immune responses by the inhibition of Th1 activation through VDR [192]. However, the role of VDR in cancer cell immunity and remodeling the TME is mainly triggered by vitamin D [193]. Hormonal vitamin D upregulates tissue-specific PD-L1 and PD-L2 surface glycoprotein expression in humans. The inhibition of VDR by its antagonist blocks PD-L1 expression, reduces the tumor burden in nude mice, and promotes anti-tumor immunity in acute myeloid leukemia, ovarian cancer, and pancreatic cancer [194]. So far, evidence on the crosstalk between the LCA/VDR axis and tumor cell immunity is sparse.

## 10. The Potential Role of FXR Agonists and Antagonists in Cancer Therapy

### 10.1. Single-Drug Therapy

**Figure 3:**
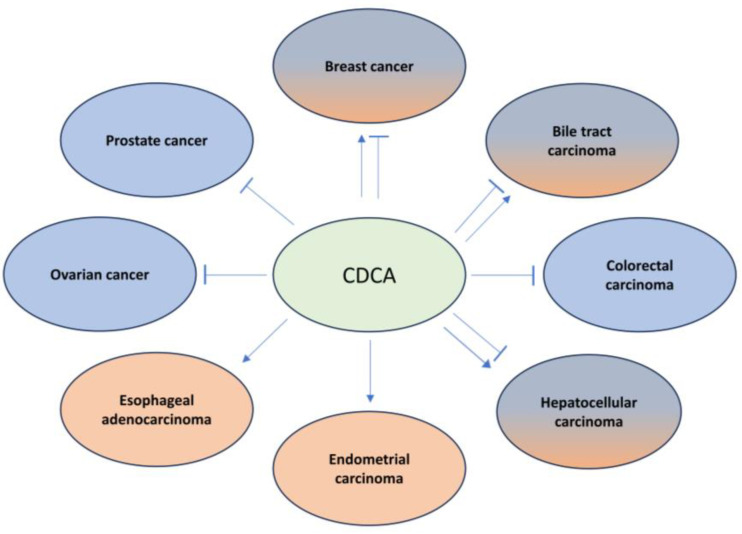
The effects of the FXR natural agonist CDCA on progression of different types of cancer including breast cancer [125,126,128], biliary tract carcinoma [196], colorectal carcinoma [199], hepatocellular carcinoma [86,201], prostate cancer [142], ovarian cancer [202], endometrial cancer [203], and esophageal adenocarcinoma [204].  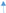 represents activation, while  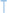 represents inhibition.

**Figure 4:**
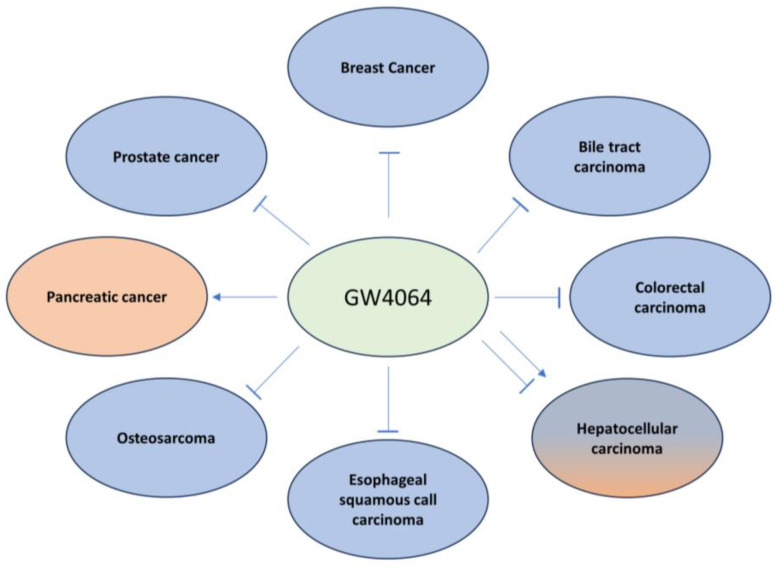
The effects of the FXR synthetic agonist GW4064 on progression of different types of cancer including breast cancer [128], biliary tract carcinoma [92], colorectal carcinoma [205], hepatocellular carcinoma [86,197], pancreatic cancer [122], prostate cancer [141], esophageal squamous cell carcinoma [110], and osteosarcoma [144].  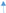 represents activation, while 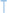 represents inhibition.

Primary and secondary BAs are endogenous ligands and nature agonists of FXR. Moreover, there are synthetic agonists including steroidal FXR agonists such as OCA and nonsteroidal FXR agonists like GW4064. CDCA, the endogenous ligand of FXR with the highest binding affinity to FXR, exerts pro- or anti-carcinogenic effects in cancer, depending on its concentration and the cellular context. As discussed before, CDCA treatment activated FXR and increased breast cancer cell proliferation and bone metastasis, while, in tamoxifen-resistant breast cancer cells, the activation of FXR by CDCA decreased cell proliferation through downregulating HER2 [125,126,128]. In biliary tract cancer (BTC), CDCA promotes cancer invasiveness through the induction of Snail and the suppression of E-cadherin [195]. However, in another study, it is observed that CDCA inhibits BTC cell growth [196]. The synthetic FXR agonist GW4064 generally exerts a tumor-suppressive function in the majority of cancer entities. However, in pancreatic cancer, GW4064-mediated FXR activation increases cell migration and invasion [122]. In HCC, the activation of FXR by GW4064 inhibits HCC growth through inhibiting the mTOR-S6K pathway, while it enhances (TGF)-β-induced EMT in HCC cells, indicating its dual role in HCC development [86,197]. The effects of CDCA and GW4064 in cancer proliferation and progression are illustrated in Figure 3 and Figure 4, respectively.

Besides CDCA and GW4064, the effects of beticholic acid (OCA), a selective agonist of FXR, and fexaramine D (Fex-D), an intestinal-restricted FXR agonist, have also been investigated in cancer cells. OCA inhibits tumor proliferation, migration, and invasion in BTC and HCC [198]. Fex-D slows the progression of tumors and significantly increases the survival rate of APC^min/+^ mice [103]. Guggulsterone is a well-studied antagonist. The inactivation of FXR by guggulsterone promotes CRC cell proliferation through increasing the expression of MMP7 [199]. Oppositely, it inhibits cell proliferation through the activation of the intrinsic apoptotic pathway in esophageal cancer [111] and HCC [200]. Additionally, it attenuates the tumor-promoting effects caused by the ectopic overexpression of FXR in pancreatic cancer and treatment with GW4064 in NSCLC [122,145].

### 10.2. Combination Therapy

These above-mentioned research data implicate that monotherapy with FXR agonists or antagonists might achieve effective anti-tumor activity. However, single-drug treatment may cause problems due to high toxicity, drug resistance, and metabolic alterations. Combination therapy is a reasonable way to overcome the shortcomings from single-drug administration. In fact, evidence demonstrates that FXR protects against cisplatin-induced kidney injury and may reduce renal side effects caused by chemotherapeutics, which implies the utility of combination therapy with an FXR agonist plus chemotherapeutic drugs [206]. Aside from the combination of FXR agonists/antagonists and anti-PD1/PD-L1 Abs, FXR agonists/antagonists combined with chemotherapy drugs, protein kinase inhibitor, and estrogen inhibitor have been proven effective in various clinical settings. Guo et al. show that GW4064 enhances the chemosensitivity of CRC to oxaliplatin by the induction of BAX/caspase-3/GSDME-mediated pyroptosis [207]. Moreover, in CRC, the combination of OCA plus nitazoxanide (NTZ), an antiparasitic drug, exerts synergistic tumor inhibition both in vitro and in vivo by co-operatively upregulating the expression of small heterodimer partner (SHP) and abrogating β-catenin expression [208]. The GW4064/cisplatin co-treatment remarkably enhances the chemosensitivity of BTC cells in vitro and inhibits the tumor growth in vivo by the upregulation of SHP expression and the downregulation of STAT3 phosphorylation [209]. Again, in BTC, OCA potentiates the anti-proliferative and pro-apoptotic effects of chemotherapeutics gemcitabine and cisplatin, representing the basis for testing OCA in clinical trials of BTC patients [196]. The activation of CDCA or GW4064 inhibits tamoxifen-resistant MCF-7 breast cancer cell growth, indicating the efficacy of the combination therapy of GW4064/tamoxifen [128].

The combination of FXR agonist PX20350 with sorafenib, a multi-kinase inhibitor against a broad range of protein kinases, reduces HCC metastasis in the lymph nodes in an orthotopic mouse xenograft model [81]. Additionally, the activation of FXR enhances hepatocyte chemoprotection and liver tumor chemoresistance against genotoxic (DNA-damaging) compounds such as doxorubicin and mitomycin C, but not against non-genotoxic drugs like paclitaxel and sorafenib [50]. Moreover, the combination of GW4064 plus acyclic retinoid (ACR), a vitamin A derivative that prevents HCC recurrence by targeting liver cancer stem cells, synergistically inhibits the growth of HCC cells by inducing apoptosis [210]. The combination of UDCA, an FXR antagonist with the targeted therapeutic drug sorafenib, shows an efficacious response in HCC by inhibiting cell proliferation and inducing apoptosis through the reactive-oxygen-species-dependent activation of ERK and the dephosphorylation of STAT3 [211]. Similarly, an in vitro study demonstrates that UDCA, in combination with gefitinib, an EGFR-tyrosine kinase inhibitor, inhibits EMT and suppresses the invasiveness of BTC cells [212]. The combination of FXR agonists/antagonist with anti-cancer drugs is summarized in Table 2.

## 11. Challenges and Future Perspectives

Numerous studies have demonstrated that FXR participates in carcinogenesis through affecting oncogenic pathways and immune pathways. FXR exerts its oncogenic or tumor-inhibitory function in a cell-dependent manner. FXR is differentially expressed in tumor cells and immune cells of the tumor microenvironment; thus, its agonists and antagonists have a distinct influence on different tumors and the TME. FXR is considered to be a target for cancer therapy. Early and current studies have yielded promising results in FXR-targeted therapy, particularly in the management of malignant neoplasms of the digestive system in which BA biosynthesis and the bacterial metabolism of BAs take place. FXR activation or inhibition is emerging as a novel therapeutic strategy that targets both malignant cells and the TME to suppress tumor growth in hepatocellular cancer (HCC) and colorectal carcinoma (CRC). Targeting FXR by using its agonists or antagonists in combination with chemotherapy drugs or immune checkpoint inhibitors largely increased therapy efficacy in HCC and CRC. The implications of these promising results extend beyond tumors in the digestive system. In breast cancer and lung cancer, FXR signaling is active, and the application of FXR agonists targeting both malignant cells and their surrounding stroma may also represent a promising avenue for the future.

However, the clinical application of FXR agonists/antagonists for cancer intervention is still challenging. So far, rare FXR agonists/antagonists have been included for clinical trials and none of them have been approved by the FDA for the treatment of cancer. The most promising FXR antagonist UDCA entered into a phase III trial for investigating its preventive role in colorectal adenoma recurrence. It turned out that UDCA treatment was associated with a non-statistically significant reduction in total colorectal adenoma recurrence [213]. Decreased long-term efficacy, drug resistance, liver toxicity, severe side effects, and metabolic disorders caused by alterations of the BA pool might be big obstacles related to the failure in FXR-targeted cancer therapy [104,208,214,215]. Recently, combination therapy with FXR agonists/antagonists plus chemotherapeutics, targeted therapeutics, or immunotherapeutic drugs, mainly anti-PD1/PD-L1 Abs, has gained more attention. Combination therapy may achieve enhanced anti-tumor activity and reduced side effects compared to monotherapy. However, there is still a long journey ahead. The optimization of concentrations of the compounds, the evaluation of the long-term effects, and the discovery of novel FXR agonists by repurposing FDA-approved drugs might be a promising approach for effectively targeting FXR in cancer [216]. Considering the fact that FXR and TGR5 are well-characterized receptors of BAs, the application of the dual FXR/TGR5 ligand might be superior to the use of a single agonist [217]. Furthermore, the mechanisms through which the FXR agonists/antagonists exert the anti-cancer effects should be studied in depth, which will be very helpful for the identification of biomarkers for the FXR-targeted cancer therapy.

## Figures and Tables

**Table 1:** The role of farnesoid X receptor (FXR) agonists/antagonists in cancer immunotherapy.

Compound	Cancer Type	Influence on Immunotherapy	Molecular Mechanism	Reference
GW4064	HCC	sensitizes anti-PD1/PD-L1 therapy		[163]
obeticholic acid	HCC		increases the secretion of CXCL16,	[164]
			IFN-γ, and NKT cell populations	
GW4064	CRC	sensitizes anti-PD1/PD-L1 therapy	increases CD8+ T cells and activates	[168]
			FXR and MAPK pathways	
GW4064	breast cancer		decreases CAF migration	[129]
UDCA	CRC	sensitizes anti-PD1/PD-L1 therapy	increases CD8+ T-cell responses,	[170]
			decreases Treg cells	
guggulsterone	LLC	upregulates PD-L1 expression	inhibits FXR and activates AKT and	[161]
			MAPK pathways	

HCC: hepatocellular carcinoma; CRC: colorectal carcinoma; LLC: Lewis lung carcinoma; UDCA: ursodeoxycholic acid; agonists: GW4064, obeticholic acid; antagonist: UDCA, guggulsterone.

**Table 2:** Combination of farnesoid X receptor (FXR) agonists/antagonists and anti-cancer drugs in cancer therapy.

Compound	Anti-Cancer Drug	Cancer Type	Mechanism	Reference
Agonist				
GW4064	oxaliplatin	CRC	enhances the chemosensitivity of cells to oxaliplatin by induction	[207]
			of BAX/caspase-3/GSDME-mediated pyroptosis in vitro	
GW4064	NTZ	CRC	synergisticly inhibits tumor growth both in vitro and in vivo by	[208]
			upregulating SHP and downregulating β-catenin	
GW4064	cisplatin	BTC	enhances chemosensitivity by upregulating SHP and	[209]
			downregulating STAT3 phosphorylation in vitro and in vivo	
OCA	gemcitabine/cisplatin	BTC	enhances the anti-proliferative and pro-apoptotic effects of	[196]
			chemotherapeutics in vitro and in vivo	
GW4064	tamoxifen	Breast Ca.	inhibits tamoxifen-resistant breast cancer cell growth in vitro	[128]
PX20350	sorafenib	HCC	reduces HCC metastasis in the lymph nodes in vivo	[81]
GW4064	doxorubicin, mitomycin C	HCC	enhances tumor chemoresistance against genotoxic drugs	[55]
GW4064	ACR	HCC	synergistically inhibits the HCC growth by inducing apoptosis in vitro	[210]
Antagonist				
UDCA	sorafenib	HCC	inhibits proliferation and induces apoptosis through ROS-dependent	[211]
			activation of ERK and dephosphorylation of STAT3 in vitro	
UDCA	gefitinib	BTC	suppresses tumor invasiveness by inhibition of EMT in vitro	[212]

CRC: colorectal carcinoma; NTZ: nitazoxanide; BTC: biliary tract carcinoma; Breast Ca.: breast cancer; HCC: hepatocellular carcinoma. ACR: acyclic retinoid; UDCA: ursodeoxycholic acid; OCA: obeticholic acid.

## Data Availability

The data presented in this study are available on request from the corresponding author.
